# Salivary RANKL/OPG and Periodontal Status Among Users of Heated Tobacco and Electronic Cigarettes Versus Non-Smokers: A Prospective Observational Study

**DOI:** 10.3390/healthcare14121797

**Published:** 2026-06-22

**Authors:** Alexandra Cornelia Teodorescu, Elena-Raluca Baciu, Irina-Georgeta Sufaru, Bogdan-Constantin Vasiliu, Alice Murariu, Sorina Mihaela Solomon

**Affiliations:** Grigore T. Popa University of Medicine and Pharmacy, 700115 Iasi, Romania; cornelia.teodorescu@umfiasi.ro (A.C.T.); ursarescu.irina@umfiasi.ro (I.-G.S.); bogdan.vasiliu@umfiasi.ro (B.-C.V.); alice.murariu@umfiasi.ro (A.M.); sorina.solomon@umfiasi.ro (S.M.S.)

**Keywords:** electronic cigarettes, heated tobacco, non-surgical periodontal therapy, osteoprotegerin, RANKL

## Abstract

**Highlights:**

**What are the main findings?**

- Both heated tobacco and electronic cigarette users exhibited significantly worse periodontal clinical parameters (periodontal probing depth and clinical attachment loss) compared with non-smokers, both at baseline and at 3 months following non-surgical periodontal therapy.
- Salivary RANKL levels were increased in heated tobacco and electronic cigarette users compared with non-smokers and remained elevated even after initial periodontal therapy.

**What are the implications of the main findings?**

- The use of these alternative tobacco products, such as heated tobacco and electronic cigarettes, is associated with a more modest response to non-surgical periodontal treatment.
- Salivary RANKL remains elevated in alternative tobacco product users after periodontal therapy, indicating a persistent shift in bone-remodeling biomarkers rather than confirming ongoing bone resorption.

**Abstract:**

**Background/Objectives:** This prospective observational cohort study aimed to evaluate the influence of heated tobacco (HT) and electronic cigarettes (ECs) on bone remodeling markers such as receptor activator of nuclear factor kappa-B ligand (RANKL) and osteoprotegerin (OPG), and periodontal status, at baseline and at 3 months after initial periodontal therapy. **Methods:** The sample comprised 236 participants (130 women, 106 men; mean age 38.96 ± 7.69 years), distributed across non-smokers (n = 72), heated tobacco/HT product users (n = 83), and electronic cigarette/EC users (n = 81). For each patient, the periodontal charting included periodontal probing depth (PPD), bleeding on probing (BOP), and clinical attachment loss (CAL). Unstimulated saliva samples were analyzed for RANKL and OPG levels. All patients underwent nonsurgical periodontal therapy (scaling and root planing). Between-group comparisons were performed using the Kruskal–Wallis test followed by Bonferroni-adjusted pairwise comparisons, while within-group changes over time were assessed using the Wilcoxon signed-rank test. To complement the primary nonparametric analyses, two-way mixed-design ANOVA and ANCOVA models adjusted for baseline values and periodontitis stage were performed as sensitivity analyses. Statistical significance was set at *p* < 0.05. **Results:** At baseline, both product user groups exhibited significantly higher PPD (*p* = 0.005) and CAL (*p* = 0.001) compared with non-smokers, with no differences between HT and EC users. Salivary RANKL levels were significantly higher in HT and EC users than in non-smokers, and OPG levels did not differ significantly. Following non-surgical periodontal therapy, all parameters improved significantly across groups (*p* < 0.001). At the 3-month follow-up, both product user groups maintained higher PPD (*p* = 0.008), CAL (*p* = 0.001), and salivary RANKL levels, compared with non-smoking individuals (*p* < 0.001). The RANKL/OPG ratio remained significantly different only for EC users compared with non-smokers (*p* < 0.001). **Conclusions:** HT and EC use were associated with differences in periodontal parameters and higher RANKL levels, while differences in the RANKL/OPG ratio were observed in EC users compared with non-smokers. Non-surgical periodontal therapy improved clinical parameters and reduced the RANKL/OPG ratio, highlighting the importance of biofilm control.

## 1. Introduction

Periodontitis is a chronic inflammatory disease that affects the tissues that support and surround the teeth. If left untreated, this disease leads to progressive and irreversible connective tissue attachment and bone loss, eventually causing tooth mobility and loss [1]. The presence of periodontal pathogens within the subgingival biofilm constitutes a necessary etiological factor for the initiation and progression of periodontitis [2]. However, only their presence is insufficient without a susceptible host immune response and additional risk factors [3].

Amongst the modifying factors, smoking is known to influence the development and progression of periodontitis [4,5,6]. Furthermore, it has been shown that patients who smoke present more severe forms of periodontitis, and the disease progression is positively correlated to the frequency and quantity of smoking [5].

Cigarette smoke contains toxic and harmful substances such as nicotine, carbon monoxide, tar, hydrogen cyanide, arsenic, benzene, and tobacco-derived nitrosamines, which are responsible for the quicker progression of periodontitis; exposure to such smoke can worsen the long-term prognosis and lead to poorer treatment results [7,8].

Emerging evidence suggests that electronic cigarettes and heated tobacco products activate similar pathophysiological mechanisms to those of conventional cigarettes, especially regarding oxidative stress, pro-inflammatory cytokine expression, and periodontal tissue damage [9,10].

HT products are new tobacco products advertised as having fewer negative health consequences [11]. They produce only aerosol, not fire, ash, or smoke, as the tobacco is heated to 350 °C rather than burned, unlike in classic cigarettes [8]. Electronic nicotine delivery systems (ENDS), including electronic cigarettes used for vaping, heat and aerosolize nicotine along with other constituents, such as flavorings, vegetable glycerin, and propylene glycol [12].

According to the World Health Organization, heated tobacco products emit many of the same toxins and carcinogens found in cigarette smoke, but in lower levels. Some compounds may occur at higher concentrations and others are novel, with uncertain implications for human health [13]. Electronic cigarettes generate harmful and potentially carcinogenic substances, deliver nicotine, and their long-term effects remain insufficiently characterized [14]. Beyond these systemic concerns, the relevance of both product types to oral and periodontal health needs more investigation.

While the negative effects of conventional cigarette smoking on periodontal healing are well established [15], the impact of non-traditional tobacco products on the reparative response following periodontal therapy remains underexplored. Emerging in vitro evidence indicates that exposure to heated tobacco product extracts significantly reduces the proliferative capacity and migratory function of human gingival fibroblasts, suppresses extracellular matrix remodeling, and delays alveolar bone recovery [16]. Similarly, electronic cigarette aerosol has been shown to induce cytotoxicity, DNA damage, oxidative stress, and apoptosis in human periodontal ligament cells and gingival fibroblasts [17,18], while also enriching periodontal pathogens and upregulating pro-inflammatory cytokines such as TNF-α and IL-6, thereby promoting a dysbiotic and pro-inflammatory oral environment that is unfavorable for tissue repair [10].

During the progression of periodontitis, there is irreversible degradation of connective tissue and alveolar bone loss. Central to this process is the RANK–RANKL–OPG system, the principal regulatory axis of bone metabolism and osteoclast-mediated resorption [19,20,21,22,23]. Although this system has also been implicated in oncological processes [24,25,26], its relevance to periodontal disease lies in regulating the balance between bone formation and resorption that results in alveolar bone loss.

The RANK–RANKL–OPG system has three interacting members. RANK is the signaling receptor for RANKL (receptor activator of nuclear factor-κB ligand), and RANKL and OPG belong to the tumor necrosis factor superfamily [27]. The binding of RANKL to RANK drives osteoclast differentiation, activity, and survival, modulating bone resorption [28]. OPG acts as a decoy receptor that binds RANKL, thereby inhibiting RANK activation, reducing osteoclastogenesis, and limiting osteoclast formation [29]. OPG additionally modulates the half-life of RANKL.

The influence of traditional smoking has been well researched in the last 20 years, with evidence confirming that smoking upregulates RANKL and downregulates OPG production in patients with periodontitis [30,31,32,33,34]. By contrast, the alternative nicotine delivery devices, such as HT and ECs, have gotten far less attention in this regard [35,36], and their impact on bone remodeling remains largely unmapped. It is not yet known whether these products sustain an unfavorable salivary RANKL/OPG profile that persists through periodontal treatment. The present study therefore aims to characterize salivary RANKL, OPG, and the RANKL/OPG ratio, as well as the clinical response to non-surgical periodontal therapy, among users of these products. The goal is to determine whether they maintain a biological environment less favorable to periodontal healing than that of non-smokers.

During the host response associated with periodontitis progression, many cytokines and immunomodulators are produced and can be detected in saliva [37]. Saliva is often preferred as a screening tool due to its ease of collection and its ability to reflect the overall inflammatory status of the mouth [38].

In light of the existing evidence, the present study aimed to evaluate short-term changes in clinical periodontal parameters and salivary bone remodeling markers following non-surgical periodontal therapy among patients with different smoking habits, with particular emphasis on heated tobacco (HT) and electronic cigarette (EC) use.

The null hypothesis was that no significant differences exist in the RANKL/OPG system and periodontal clinical outcomes among non-smokers, HT users, and EC users following periodontal therapy.

## 2. Materials and Methods

### 2.1. Research Design and Criteria for Patient Selection

This is a prospective observational study with repeated measurements at baseline and 3 months across different study groups. Participants were recruited consecutively from new patients presenting for periodontal evaluation and treatment at the Department of Periodontology, “Grigore T. Popa” University of Medicine and Pharmacy, Iași, Romania. No online or advertisement-based recruitment was used. All participants were identified during routine clinical attendance and invited to take part if they met the eligibility criteria. A total of 251 participants were enrolled, of whom 236 completed the 3-month follow-up and were included in the final analysis.

The study protocol was approved by the Ethics Committees of Grigore T. Popa University of Medicine and Pharmacy in Iasi (approval number 5328/8 March 2018). All procedures were carried out in accordance with the ethical principles outlined in the Declaration of Helsinki for research involving human subjects. All participants received detailed information regarding the purpose and procedures of the study. After agreeing to take part, they were instructed to carefully review and sign the informed consent form.

Sample size estimation was performed using G*Power software (version 3.1, Heinrich Heine University Düsseldorf, Germany), applying an F-test for a one-way ANOVA with three independent groups as the statistical model. A medium effect size (*f* = 0.25) was selected based on Cohen’s classification of effect sizes; in the absence of prior studies directly characterizing salivary RANKL/OPG differences among heated tobacco users, electronic cigarette users, and non-smokers, the medium convention was considered a conservative and appropriate assumption. A conventional two-sided significance level of *α* = 0.05 was applied, and a statistical power of 90% (1 − *β* = 0.90) was chosen over the more commonly used 80% threshold to minimize the risk of Type II error, particularly given the exploratory nature of the biomarker outcome comparisons. Under these parameters, the minimum required sample size was calculated to be 207 participants (69 per group). A dropout rate of 15–20% was anticipated based on attrition rates reported in comparable periodontal studies [39,40], and a target enrollment of approximately 240–250 participants was therefore planned. In total, 251 participants were enrolled, of whom 236 (94.0%) completed the 3-month follow-up and were included in the final analysis, confirming that the attained sample size preserved the intended statistical power.

### 2.2. The Study Group

Inclusion criteria consisted of [40]:

- Non-smokers, heated tobacco users, or electronic cigarette users (with nicotine);
- Diagnosis of periodontitis—stages I, II, and III;
- Age between 18 and 55 years;
- Presence of at least 20 natural teeth.

Patients diagnosed with stage IV periodontitis were not subjected to this study, as this stage signifies a distinct clinical condition characterized by significant tooth loss, functional impairment, and complex therapeutic requirements. Furthermore, bone metabolism in stage IV disease is markedly altered, and salivary biomarkers such as RANKL and OPG may indicate chronic remodeling processes rather than acute inflammatory responses.

The exclusion criteria consisted of [40,41]:

- Ages below 18 or above 55 years;
- Burned tobacco users or mixed users (burned tobacco and EC/HT) with at least one traditional cigarette smoked in the last year;
- Gingivitis;
- Chronic systemic diseases that could influence periodontal status, including diabetes, immunity disorders, leukemia, osteopenia, or osteoporosis;
- Chronic medication administration;
- Pregnant and lactating women;
- Antibiotics taken within the previous 6 months;
- Taking anti-inflammatory drugs in the past 4 weeks;
- Receiving periodontal therapy within the past 12 months;
- Having fewer than 20 teeth or wearing fixed orthodontic appliances.

None of the enrolled participants were undergoing orthodontic treatment with clear aligners at the time of periodontal examination. Participants were divided into three groups according to smoking status: non-smokers, heated tobacco (HT) users, and electronic cigarette (EC) users.

Furthermore, the periodontal status of each patient was established according to the 2018 AAP/EFP new classification [5]: Stage I periodontitis—with clinical interdental attachment loss of 1–2 mm, maximal probing depth of 4 mm, no teeth lost due to periodontitis and a pattern of horizontal bone loss; Stage II—3–4 mm interproximal clinical attachment loss (CAL), a maximum of 5 mm probing depth, horizontal bone loss and no periodontal related tooth loss; Stage III—≥5 mm interdental CAL at the site of greatest loss, probing depth of at least 6 mm, a maximum of 4 periodontitis-related teeth lost; can associate vertical bone and furcation involvement class II or III;

All patients underwent phase 1 periodontal treatment (non-surgical scaling and root planing in two steps—1 dental arch per week, with manual and ultrasonic instruments) and were examined a second time at 3 months after completion of the treatment [42,43,44].

The data collected from the patients were organized as follows:

- Demographic variables, such as age and gender;
- The smoking status: non-smokers, heated tobacco, or E-cigarette users through a medical questionnaire;
- Periodontal charting at baseline (T0) and three months post-treatment (T1), which included periodontal probing depth (PPD), bleeding on probing (BOP), and clinical attachment loss (CAL);
- Periodontitis stages;
- Intraoral photographs from before, during, and after treatment.

### 2.3. Clinical Evaluation and Saliva Collection

All the patients were evaluated at baseline and at the 3-month follow-up. From a periodontal standpoint, we examined bleeding on probing (BOP), pocket probing depth (PPD), and clinical attachment loss (CAL).

BOP was determined with the help of a periodontal probe (North Carolina or CP/15) by gently probing every lateral aspect of each tooth. BOP was calculated as the percentage of bleeding sites reported relative to the number of examined sites [45].

Using the same periodontal probes, PPD was calculated for each tooth as the distance between the junctional epithelium and the gingival margin, and CAL—the distance between the junctional epithelium and the CEJ (cementum-enamel junction) [46].

Before the clinical examination, two experienced examiners (A.C.T. and B.C.V.) were calibrated to assess BOP, PPD, and CAL. Calibration was performed on a group of patients not included in the main study. Inter-examiner reliability was evaluated using the intraclass correlation coefficient (ICC) for PPD and CAL and Cohen’s kappa statistic for BOP. Excellent agreement between examiners was observed (ICC > 0.90; *κ* > 0.80).

Unstimulated saliva samples were collected from each eligible patient at baseline and 3 months after periodontal therapy. After a 1 h period during which patients did not eat, drink, or brush their teeth, 3 to 5 mL of unstimulated saliva was collected in sterile urine containers over a maximum of 5 min. The samples were then stored at −80 °C until further analysis [47,48,49].

Saliva samples were processed and analyzed every 6 months during the study to prevent long storage periods and sample deterioration [50,51].

When it was time for analysis, the saliva samples were thawed to room temperature and then centrifuged at 1000× *g* for 20 min [52]. The samples were processed and analyzed according to the manufacturer’s specifications, using the Human RANKL (Receptor Activator of Nuclear Factor Kappa-B Ligand) ELISA kit and the Human OPG (Osteoprotegerin) ELISA kit from Elabscience (Houston, TX, USA).

### 2.4. Statistical Analysis

All statistical analyses were carried out using IBM SPSS Statistics software (version 26.0; IBM Corp., Armonk, NY, USA). Descriptive statistics were presented as counts (N) and percentages (%) for categorical variables, and as mean ± standard deviation or median (25th–75th percentiles) for continuous variables.

The distribution of continuous variables was assessed using the Shapiro–Wilk test. Because the data deviated from normality, nonparametric statistical methods were employed for further analyses. Differences between baseline and 3-month follow-up values within the same group were analyzed using the Wilcoxon signed-rank test. Comparisons between independent groups were performed using the Kruskal–Wallis test, followed by Bonferroni-adjusted pairwise comparisons.

To complement the nonparametric analyses, a two-way mixed-design ANOVA was performed for each outcome variable, with smoking group (non-smokers, HT users, EC users) as the between-subject factor and time (baseline, 3 months) as the within-subject factor, to assess the main effects of group and time and their interaction. Post hoc pairwise comparisons were conducted with Bonferroni correction. Additionally, an analysis of covariance (ANCOVA) was performed for each 3-month outcome, with smoking group as the fixed factor and both the corresponding baseline value and periodontitis stage as covariates, to assess between-group differences after adjustment for baseline disease severity. The significance threshold was set at *p* < 0.05 for all analyses.

## 3. Results

**Figure 1:**
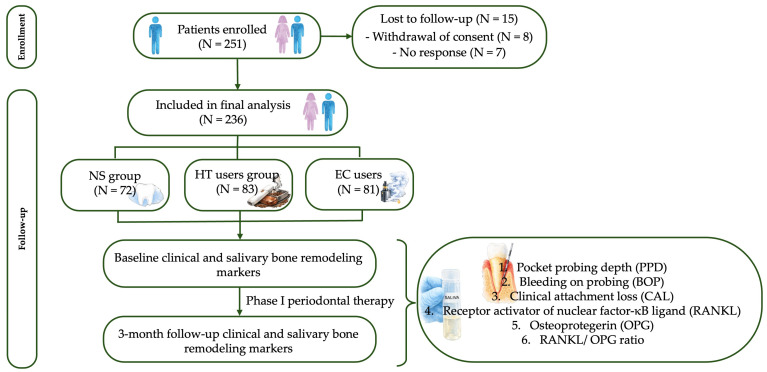
Schematic representation of the study design.

**Figure 2:**
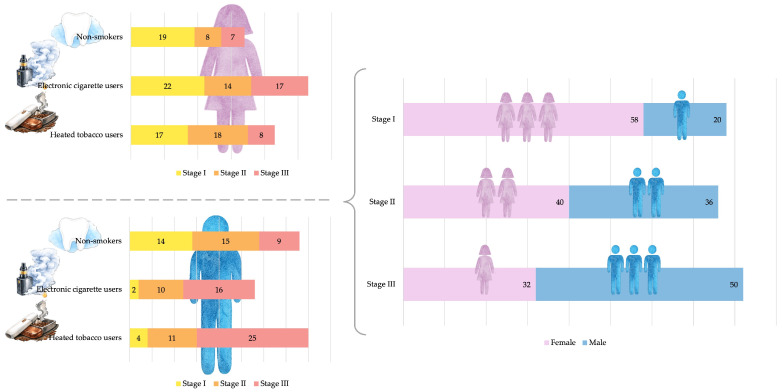
Gender-based distribution of periodontitis stages according to smoking status.

The study included 236 patients who completed the three-month follow-up, from a total of 251 initially recruited (Figure 1). Of the 236 participants, 130 were females and 106 were males, with an average age of 38.96 ± 7.69 years. Regarding periodontal status, most patients were diagnosed with stage III periodontitis (Table 1), of whom 60.9% were males (Figure 2).

All groups showed improvements in clinical parameters following non-surgical periodontal therapy, with significant reductions in PPD, BOP, and CAL observed within each group (*p* < 0.001). At baseline, statistically significant differences between groups were identified for PPD (*p* = 0.005) and CAL (*p* = 0.001), whereas BOP did not differ significantly (*p* = 0.178) (Table 2).

At 3 months, between-group differences persisted for PPD (*p* = 0.008) and CAL (*p* = 0.001), while BOP remained comparable across groups (*p* = 0.475). No significant differences were observed between HT and EC users at either time point (Table 2).

Salivary biomarkers showed consistent changes following non-surgical periodontal therapy, with decreases in RANKL levels and increases in OPG levels observed within all groups (*p* < 0.001), resulting in a reduction in the RANKL/OPG ratio (Table 3).

At baseline, statistically significant between-group differences were identified for RANKL (*p* < 0.001), while no significant differences were observed for OPG (*p* = 0.116). Pairwise comparisons indicated differences between non-smokers and both product use groups, with no significant differences between HT and EC users. At 3 months, between-group differences remained significant for RANKL (*p* < 0.001), whereas OPG values did not differ significantly between groups (*p* = 0.264). Post hoc comparisons showed differences between non-smokers and EC users in the RANKL/OPG ratio, whereas no differences were observed between HT and EC users or between HT users and non-smokers (Table 3).

Two-way mixed-design ANOVA confirmed a significant main effect of time for all outcome variables (*p* < 0.001), consistent with the effectiveness of non-surgical periodontal therapy across all groups. A significant main effect of smoking group was observed for PPD (F = 4.752, *p* = 0.010), CAL (F = 6.158, *p* = 0.003), RANKL (F = 9.635, *p* < 0.001), and the RANKL/OPG ratio (F = 8.553, *p* < 0.001), but not for BOP (F = 2.501, *p* = 0.084) or OPG (F = 1.676, *p* = 0.189). Notably, a significant Group × Time interaction was identified for salivary RANKL (F = 10.027, *p* < 0.001, η^2^p = 0.079) and the RANKL/OPG ratio (F = 5.983, *p* = 0.003, η^2^p = 0.049), indicating that the magnitude of the treatment-associated reduction in these biomarkers differed significantly across groups (Table 4).

ANCOVA, adjusting for baseline values and periodontitis stage, demonstrated that between-group differences in clinical parameters (PPD, BOP, CAL) were no longer statistically significant after covariate adjustment (all *p* > 0.05), suggesting that the clinical differences observed in the unadjusted analyses were substantially explained by baseline disease severity. In contrast, the group effect for salivary RANKL remained significant after adjustment (F = 5.180, *p* = 0.006), with both HT users (*p* = 0.023) and EC users (*p* = 0.002) showing higher adjusted RANKL levels at 3 months compared with non-smokers. Group effects for OPG and the RANKL/OPG ratio did not reach significance after adjustment (Table 5).

## 4. Discussion

To the best of our knowledge, this study is among the first to discuss the influence of alternative tobacco products (heated tobacco and electronic cigarettes) on the dynamics of the RANKL/OPG system and periodontal status during phase 1 periodontal therapy.

As presented, the study population consisted of 72 non-smokers (30.5%), 83 heated tobacco users (35.2%), and 81 electronic cigarette users (34.3%), reflecting a balanced distribution across the smoking categories. Regarding periodontitis severity, 33.1% of participants were classified as Stage I, 32.2% as Stage II, and 34.7% as Stage III.

The findings also revealed a gender-based distribution in tobacco product preferences, with 40% of female participants opting for ECs and 38% of male participants preferring HT products. The female preference for ECs is consistent with reports that women associate e-cigarettes with weight control, mood management, and appetite regulation [53], and that they prefer slimmer, cigarette-like devices [54]. The male preference for HT products may reflect gender differences in nicotine sensitivity, with men reported to be more sensitive to nicotine dosing and thus favoring the more precise nicotine delivery of heated tobacco [53], as well as a stronger motivation based on the perceived lower harm of these products [54]. These observations suggest that novel tobacco product adoption may be shaped by distinct behavioral and psychosocial motivations across sexes, and a better understanding of such patterns could inform more targeted smoking-cessation strategies for users of heated tobacco and electronic cigarettes.

The finding that both EC and HT users presented with significantly greater PPD (*p* = 0.005) and CAL (*p* = 0.001) compared with non-smoking controls aligns with a growing body of evidence demonstrating that novel tobacco and nicotine products are not without consequence to periodontal tissues. A cross-sectional study by Alnufaiy et al. [55] comparing EC users to non-smokers reported significantly greater PPD and CAL in the EC group (*p* < 0.05).

Beyond statistical significance, the magnitude of the observed differences merits interpretation. The between-group differences in PPD were small (approximately 0.2 mm), but statistically significant given the sample size. In contrast, the differences in CAL were more substantial, reaching approximately 1.3 mm between non-smokers and EC users at baseline, and are more likely to be clinically relevant, indicating greater attachment loss in users of novel nicotine-delivery products. This suggests that the periodontal impact of these products is better reflected by cumulative attachment damage than by pocket depth at a single time point.

The absence of significant differences in BOP across the three groups, both at baseline and at 3 months, is clinically relevant and should be interpreted in the context of the present study design evaluating the periodontal effects of alternative tobacco products. While conventional cigarette smoking is known to suppress bleeding on probing through nicotine-induced vasoconstriction and to promote periodontal attachment loss [32], the present study focused on the less characterized periodontal effects of HT and ECs.

The present study found that both heated tobacco and electronic cigarette users exhibit significantly higher salivary levels of RANKL and RANKL/OPG ratios (*p* < 0.001) compared with non-smokers. Notably, no significant difference was found between the two alternative product-use groups, suggesting comparable immunological effects on the bone remodeling system despite different delivery mechanisms.

These findings align with previous studies published throughout the years. Ibraheem et al. [35] measured the RANKL and OPG levels in gingival crevicular fluid (GCF) from 120 men and found significantly higher GCF RANKL levels among cigarette smokers, water-pipe smokers, and electronic-nicotine-delivery-systems (ENDS) users (*p* < 0.001), compared with non-smokers. Although measured in GCF rather than saliva, this supports the association between nicotine-containing products and elevated RANKL, consistent with our findings.

One unexpected finding in our study was the lack of a significant difference in salivary OPG levels between product users and non-smokers. This is supported by Behfarnia et al. [56], who reported that OPG levels in saliva, GCF, and serum did not differ significantly between chronic periodontitis patients and periodontally healthy non-smokers. OPG levels may remain stable in some populations, despite elevated RANKL levels, or be less responsive than RANKL to smoking-induced immunological changes. Some older studies report the opposite: lower OPG levels in smokers than in non-smokers, in saliva [31] or serum [30].

Non-surgical periodontal therapy (scaling and root planing) is highly effective in improving periodontal parameters in both smokers and non-smokers, as shown in Table 2 (*p* < 0.001). This observation is well documented in the recent literature [40,57,58]. Moreover, EFP clinical guidelines state that nonsurgical periodontal treatment should be implemented for all patients with periodontitis, regardless of disease stage [42].

At 3 months following non-surgical periodontal therapy, both HT and EC users continued to exhibit significantly higher PPD (*p* = 0.008) and CAL (*p* = 0.001) values compared with non-smoking controls, while no significant differences were detected between the two product use groups. These findings indicate that the use of these alternative tobacco products is associated with a more modest response to non-surgical periodontal treatment, a pattern observed in a recent systematic review and meta-analysis. Tattar et al. [59] reported that following periodontal interventions, e-cigarette users had significantly higher residual PPD compared with non-smokers, reinforcing the notion that nicotine-containing aerosols may compromise periodontal tissue repair.

The comparable PPD and CAL values between heated tobacco users and electronic cigarette users at 3 months further suggest that these two product categories exert comparable effects on periodontal healing. This observation is supported by a recent systematic review by Dipalma et al. [10], which concluded that both electronic cigarettes and heated tobacco products are associated with delayed periodontal healing and treatment outcomes.

Salivary RANKL levels remained significantly elevated in both HT and EC users compared with non-smokers (*p* < 0.001) at the 3-month follow-up, with no significant difference between the two product use groups. This persistent elevation of RANKL despite clinical improvement after non-surgical periodontal therapy may reflect continued alterations in bone-remodeling-related pathways. However, salivary biomarker levels alone do not provide direct evidence of ongoing osteoclast activity or alveolar bone resorption.

A particularly noteworthy finding at 3 months was the differential behavior of the RANKL/OPG ratio across groups. Electronic cigarette users maintained a significantly higher RANKL/OPG ratio compared with non-smokers, with statistical significance only for the EC group (*p* < 0.001). Heated tobacco users occupied an intermediate position, with no significant difference from either non-smokers or electronic cigarette users. This pattern suggests that electronic cigarette use may be associated with a more sustained elevation of RANKL/OPG ratio than heated tobacco products, following periodontal therapy. One possible explanation is the difference in aerosol composition between the two product types: e-cigarette aerosol contains propylene glycol and vegetable glycerin as primary humectants, which have been shown to induce oxidative stress, DNA damage, and upregulation of inflammatory cytokines in human gingival fibroblasts [18,60]. It should be noted that, in the absence of quantified consumption data, a contribution of differing exposure intensity between the two product groups cannot be excluded.

The two-way mixed ANOVA revealed a significant Group × Time interaction for salivary RANKL and the RANKL/OPG ratio, indicating that the trajectories of these biomarkers over the treatment period differed across groups—not merely their absolute levels. Furthermore, ANCOVA demonstrated that, while between-group differences in clinical parameters (PPD and CAL) were largely attenuated after adjustment for baseline disease severity and periodontitis stage, the group effect on salivary RANKL persisted independent of these covariates. This finding suggests that the elevated RANKL levels observed in HT and EC users are not simply a reflection of more advanced baseline disease, but may represent a genuine, product-associated alteration in the bone remodeling environment.

Moreover, the three-month follow-up period captures the early post-therapy response and cannot establish whether these biomarker differences translate into measurable differences in long-term clinical or radiographic disease progression. Longer-term studies incorporating radiographic outcomes are needed to determine the clinical significance of these associations.

Clinicians should be aware that users of heated tobacco products and electronic cigarettes may present less favorable periodontal clinical parameters, elevated bone remodeling biomarkers, and a reduced response to non-surgical periodontal therapy compared with non-smokers.

### Study Limitations

This study has several limitations that should be acknowledged in accordance with the STROBE guidelines for observational studies. Regarding selection bias, participants were recruited consecutively from a single university clinic, introducing an inherent selection toward individuals already seeking dental care. Because participation was voluntary, individuals with particularly poor periodontal health or heavy tobacco dependence may have been less likely to enroll or complete follow-up, which could underestimate the true periodontal impact of these products.

With respect to attrition bias, 15 of the 251 enrolled participants (6.0%) did not complete the 3-month follow-up. As no systematic data were available to characterize these individuals, it cannot be determined whether dropout was random or different across groups. If participants with more severe disease or heavier use were more likely to withdraw, the 3-month outcomes may reflect a more favorable treatment response than would be seen in the full cohort.

The findings should also be interpreted in light of confounding. The distribution of periodontitis stages differed significantly across groups (chi-square *p* = 0.034), with non-smokers presenting proportionally milder disease at baseline. After adjustment for baseline severity and periodontitis stage, ANCOVA showed that between-group differences in clinical parameters were substantially attenuated, indicating that stage imbalance contributed to the observed clinical differences. The group effect for salivary RANKL, however, persisted. Because a fully adjusted multivariable model incorporating all relevant covariates was not performed, residual confounding from unmeasured variables (including oral hygiene, diet, socioeconomic status, psychosocial stress, and individual immune variation) cannot be excluded.

Exposure to tobacco products was assessed by structured questionnaire and self-report without biochemical verification (salivary or urinary cotinine), so some exposure misclassification cannot be excluded. Detailed data on frequency, duration, cumulative exposure, and device characteristics were also unavailable, and individual variability in nicotine metabolism and host immune response may have further influenced the observed associations.

A conventional cigarette smoker group was not included, as the periodontal consequences of traditional smoking are already well established. Our results therefore cannot be compared head-to-head with conventional smoking but may guide future work. Similarly, salivary biomarkers provide a non-invasive reflection of bone-remodeling and inflammatory processes but cannot fully characterize local periodontal tissue events.

Finally, the single-center design may limit generalizability. Adequately powered, multicenter periodontal studies with larger cohorts of heated-tobacco and electronic-cigarette users are needed to clarify whether these products independently contribute to periodontitis progression and to define their relationship with the RANKL/OPG pathway.

## 5. Conclusions

Both HT and EC users exhibited significantly worse periodontal clinical parameters (PPD and CAL) compared with non-smokers, both at baseline and at 3 months following non-surgical periodontal therapy, suggesting that these alternative tobacco products are associated with greater periodontal tissue destruction and an attenuated therapeutic response.

Salivary RANKL levels were increased in product use groups compared with non-smokers, while the RANKL/OPG ratio differed significantly only in electronic cigarette users, supporting an association with a pro-resorptive biomarker profile.

## Figures and Tables

**Table 1:** Characteristics of study participants (N = 236).

Variable	*Gender*	
	Male	106 (44.9%)
	Female	130 (55.1%)
	*Age*	
	38.96 ± 7.69 years (20–50)	
	*Smoking status*	
	Non-smokers	72 (30.5%)
	Heated tobacco users	83 (35.2%)
	Electronic cigarettes users	81 (34.3%)
	*Periodontitis*	
	Stage I	78 (33.1%)
	Stage II	76 (32.2%)
	Stage III	82 (34.7%)

Data are presented as counts and percentages or mean ± standard deviation (minimum–maximum), as appropriate.

**Table 2:** Clinical periodontal parameters according to the smoking status, at baseline and 3 months after periodontal therapy.

Clinical Parameters		Smoking Status			*p*-Value (Between Group)	
		Non-Smokers (N = 72)	Heated Tobacco Users (N = 83)	Electronic Cigarette Users (N = 81)		
PPD (mm)	Baseline	3.83 (3.2–4.16)	4.08 (3.83–4.75)	4 (3.5–4.83)	*0.005*	NS vs. HT = *0.021*
						NS vs. EC = *0.008*
						HT vs. EC > 0.999
	3 months	2.41 (2.08–2.91)	2.58 (2.41–3.08)	2.66 (2.33–3.16)	*0.008*	NS vs. HT = *0.033*
						NS vs. EC = *0.013*
						HT vs. EC > 0.999
	*p*-value (within group)	*<0.001*	*<0.001*	*<0.001*	-	-
BOP (%)	Baseline	54 (42–67.5)	54 (46–67.5)	58 (48–75)	0.178	-
	3 months	15.5 (6–26.5)	16 (8–26.5)	16 (8–27)	0.475	-
	*p*-value (within group)	*<0.001*	*<0.001*	*<0.001*	-	-
CAL (mm)	Baseline	1.75 (1.45–3.2)	2.75 (1.95–4.66)	3.08 (1.91–4.83)	*0.001*	NS vs. HT = *0.015*
						NS vs. EC = *0.001*
						HT vs. EC > 0.999
	3 months	1.58 (0.91–2.79)	2.16 (1.5–3.87)	2.21 (1.58–3.75)	*0.001*	NS vs. HT = *0.008*
						NS vs. EC = *0.002*
						HT vs. EC > 0.999
	*p*-value (within group)	*<0.001*	*<0.001*	*<0.001*	-	-

Data are presented as median (and 25th–75th percentiles). Differences between groups were assessed using the Kruskal–Wallis test followed by Bonferroni-adjusted pairwise comparisons, whereas differences within groups were assessed using the Wilcoxon signed-rank test. Italicized *p*-values indicate statistical significance (*p* < 0.05). PPD—periodontal probing depth; BOP—bleeding on probing; CAL—clinical attachment loss; NS—non-smokers; HT—heated tobacco users; EC—electronic cigarette users.

**Table 3:** Salivary biomarkers according to the smoking status, at baseline and 3 months after periodontal therapy.

Salivary Parameters		Smoking Status			*p*-Value (Between Group)	
		Non-Smokers (N = 72)	Heated Tobacco Users (N = 83)	Electronic Cigarettes Users (N = 81)		
RANKL (pg/mL)	Baseline	23 (18.5–41)	35 (21–52)	42 (29–61)	*<0.001*	NS vs. HT = *0.029*
						NS vs. EC *< 0.001*
						HT vs. EC = 0.206
	3 months	19 (13.5–35)	29 (17–40.5)	34 (24–47)	*<0.001*	NS vs. HT = *0.030*
						NS vs. EC *< 0.001*
						HT vs. EC = 0.259
	*p*-value (within group)	*<0.001*	*<0.001*	*<0.001*	-	-
OPG (pg/mL)	Baseline	55 (43–63)	55 (49–67)	52 (46–61)	0.116	-
	3 months	62 (47–70.5)	62 (54.5–73)	56 (54–68)	0.264	-
	*p*-value (within group)	*<0.001*	*<0.001*	*<0.001*	-	-
RANKL/ OPG ratio	Baseline	0.53 (0.36–0.7)	0.61 (0.45–0.87)	0.76 (0.51–1.22)	*<0.001*	NS vs. HT = 0.078
						NS vs. EC *< 0.001*
						HT vs. EC = *0.043*
	3 months	0.33 (0.27–0.57)	0.43 (0.31–0.7)	0.57 (0.39–0.76)	*<0.001*	NS vs. HT = 0.087
						NS vs. EC *< 0.001*
						HT vs. EC = 0.067
	*p*-value (within group)	*<0.001*	*<0.001*	*<0.001*	-	-

Data are presented as median (and 25th–75th percentiles). Differences between groups were assessed using the Kruskal–Wallis test followed by Bonferroni-adjusted pairwise comparisons, whereas differences within groups were assessed using the Wilcoxon signed-rank test. Italicized *p*-values indicate statistical significance (*p* < 0.05). RANKL—receptor activator of nuclear factor-κB ligand; OPG—osteoprotegerin; NS—non-smokers; HT—heated tobacco users; EC—electronic cigarette users.

**Table 4:** Two-way mixed-design ANOVA results for clinical and salivary parameters according to smoking status and time.

Outcome	Smoking Group (Between-Subject)	Time (Within-Subject)	Group × Time Interaction	Post hoc Pairwise Comparisons (Bonferroni-Corrected)
PPD	F = 4.752	F = 1649.592	F = 2.268	NS vs. EC: *p* = *0.017*
	*p = 0.010*	*p < 0.001*	*p* = 0.106	NS vs. HT: *p* = 0.098
	η^2^p = 0.039	η^2^p = 0.876	η^2^p = 0.019	HT vs. EC: *p* > 0.999
BOP	F = 2.501	F = 1785.797	F = 0.136	All comparisons non-significant
	*p* = 0.084	*p < 0.001*	*p* = 0.873	
	η^2^p = 0.021	η^2^p = 0.885	η^2^p = 0.001	
CAL	F = 6.158	F = 712.498	F = 2.088	NS vs. EC: *p < 0.001*
	*p = 0.003*	*p < 0.001*	*p* = 0.126	NS vs. HT: *p = 0.001*
	η^2^p = 0.050	η^2^p = 0.754	η^2^p = 0.018	HT vs. EC: *p* = 0.980
RANKL	F = 9.635	F = 652.439	F = 10.027	NS vs. HT: *p = 0.003*
	*p < 0.001*	*p* < *0.001*	*p* < *0.001*	NS vs. EC: *p < 0.001*
	η^2^p = 0.076	η^2^p = 0.737	η^2^p = 0.079	HT vs. EC: *p = 0.008*
OPG	F = 1.676	F = 991.010	F = 0.215	All comparisons non-significant
	*p* = *0.189*	*p < 0.001*	*p* = 0.806	
	η^2^p = 0.014	η^2^p = 0.810	η^2^p = 0.002	
RANKL/OPG ratio	F = 8.553	F = 575.540	F = 5.983	NS vs. HT: *p = 0.023*
	*p < 0.001*	*p < 0.001*	*p* = 0.003	NS vs. EC: *p < 0.001*
	η^2^p = 0.068	η^2^p = 0.712	η^2^p = 0.049	HT vs. EC: *p = 0.008*

F-values and partial eta squared (η^2^p) are reported for each source of variation. Post hoc pairwise comparisons were conducted with Bonferroni correction. Italicized *p*-values indicate statistical significance (*p* < 0.05). NS—non-smokers; HT—heated tobacco users; EC—electronic cigarette users; η^2^p—partial eta squared; PPD—periodontal probing depth; BOP—bleeding on probing; CAL—clinical attachment loss; RANKL—receptor activator of nuclear factor-κB ligand; OPG—osteoprotegerin.

**Table 5:** Analysis of covariance (ANCOVA) for clinical and salivary parameters at 3 months, adjusted for baseline values and periodontitis stage.

Outcome	R^2^	Smoking Group (F, *p*)	Baseline Value (F, *p*)	Periodontitis Stage (F, *p*)	Adjusted Pairwise Contrasts (Bonferroni-Corrected)
PPD	0.723	F = 2.324 *p* = 0.100	F = 191.2 *p < 0.001*	F = 32.6 *p < 0.001*	HT vs. NS: *p =* 0.115
					EC vs. NS: *p =* 0.722
					HT vs. EC: *p = 0.043*
BOP	0.379	F = 0.630 *p* = 0.534	F = 50.4 *p* < *0.001*	F = 7.8 *p = 0.001*	HT vs. NS: *p* = 0.830
					EC vs. NS: *p* = 0.421
					HT vs. EC: *p* = 0.285
CAL	0.949	F = 0.698 *p* = 0.499	F = 967.6 *p < 0.001*	F = 3.2 *p = 0.041*	HT vs. NS: *p* = 0.314
					EC vs. NS: *p* = 0.973
					HT vs. EC: *p* = 0.312
RANKL	0.975	F = 5.180 *p = 0.006*	F = 3027.3 *p* < *0.001*	F = 23.9 *p* < *0.001*	HT vs. NS: *p = 0.023*
					EC vs. NS: *p = 0.002*
					HT vs. EC: *p* = 0.320
OPG	0.975	F = 0.751 *p* = 0.473	F = 6709.4 *p* < *0.001*	F = 19.3 *p < 0.001*	HT vs. NS: *p* = 0.228
					EC vs. NS: *p* = 0.626
					HT vs. EC: *p* = 0.454
RANKL/OPG ratio	0.942	F = 0.891 *p* = 0.412	F = 2104.2 *p* < *0.001*	F = 2.5 *p* = 0.087	HT vs. NS: *p* = 0.214
					EC vs. NS: *p* = 0.783
					HT vs. EC: *p* = 0.321

The dependent variable for each model was the corresponding 3-month outcome. Covariates: baseline value of the respective parameter and periodontitis stage. Adjusted contrasts are derived from the ANCOVA model with Bonferroni correction. Italicized *p*-values indicate statistical significance (*p* < 0.05). NS—non-smokers; HT—heated tobacco users; EC—electronic cigarette users; R^2^—coefficient of determination; PPD—periodontal probing depth; BOP—bleeding on probing; CAL—clinical attachment loss; RANKL—receptor activator of nuclear factor-κB ligand; OPG—osteoprotegerin.

## Data Availability

The original contributions presented in this study are included in the article. Further inquiries can be directed to the corresponding author.

## Abbreviations

The following abbreviations are used in this manuscript:

BOP	Bleeding on probing
CAL	Clinical attachment loss
EC	Electronic cigarette
ENDS	Electronic nicotine delivery systems
GCF	Gingival crevicular fluid
HT	Heated tobacco
OPG	Osteoprotegerin
PPD	Periodontal probing depth
RANKL	Receptor activator of nuclear factor-κB ligand
WHO	World Health Organization
